# A disease-relevant mutation of SPOP highlights functional significance of ATM-mediated DNA damage response

**DOI:** 10.1038/s41392-020-00381-7

**Published:** 2021-01-15

**Authors:** Mingming Xiao, Joshua S. Fried, Jinlu Ma, Yang Su, Rebecca J. Boohaker, Qinghua Zeng, Yaqi Mo, Fanbiao Meng, Rong Xiang, Bo Xu

**Affiliations:** 1 Department of Biochemistry and Molecular Biology, Key Laboratory of Breast Cancer Prevention and Therapy, Ministry of Education, Tianjin Medical University Cancer Institute and Hospital, National Clinical Research Center for Cancer, Key Laboratory of Cancer Prevention and Therapy, Tianjin’s Clinical Research Center for Cancer, Tianjin, 300060 China; 2 Department of Oncology, Southern Research Institute, Birmingham, AL 35205 USA; 3 Cell Biology Program, University of Alabama at Birmingham, Birmingham, AL 35205 USA; 4 Department of Radiation Oncology, First Affiliated Hospital, Xian Jiaotong University, Xi’an, China; 5 Department of Biochemistry and Molecular Biology, Nankai University School of Medicine, Tianjin, China; 6 Center for Intelligent Oncology, Chongqing University Cancer Hospital, Chongqing University School of Medicine, Chongqing, 400030 China

**Keywords:** Cancer, Diseases

**Dear Editor,**

Accumulating evidence supports that, as a critical genome guardian mechanism, the DNA damage response (DDR) is a barrier to early tumorigenesis. As a crucial DDR pathway, Ataxia Telangiectasia Mutated (ATM)-mediated phosphorylation of downstream targets is essential for activation of cell cycle checkpoints, DNA repair, and programed cell death in the presence of DNA damage.^1^ Despite extensive biochemical and cellular studies on ATM phosphorylation, these phosphorylation sites are rarely mutated in cancers, challenging the pathophysiological relevance of ATM-mediated phosphorylation in the disease setting.

Prostate cancer is one of the most common cancers in men. Genomic studies have shown that the gene encoding Speckle type Poz Protein (SPOP), an E3 ubiquitin ligase adaptor, is among the most frequently mutated in prostate cancer.^2^ Recent studies have shown that SPOP plays a critical role in the DDR, and clinical data support that SPOP mutations are associated with genomic instability.^3^ Among SPOP mutation sites, we found that the Serine 119 mutation to asparagine (S119N), one of the clinically relevant mutations, resulted in a radiosensitive phenotype. S119N mutated prostate cancer cells showed hypersensitivity to IR (Fig. 1a, Supplementary Fig. S1a–c) and a loss of the IR-induced G2/M checkpoint (Fig. 1b, Supplementary Fig. S1d). We also assessed if the faulty DNA repair mechanics caused by the S119 mutation had an impact on genomic instability, measured by the number of micronuclei in response to IR. We found that S119N cells had a significant increase in the amount of micronuclei positive cells after IR (Fig. 1c, Supplementary Fig. S1e). We also found cells with S119N have persistent γ-H2AX accumulation after IR (Fig. 1d, Supplementary Fig. S1f). In nonmalignant prostate epithelial cells (Supplementary Fig. S1g), induction of S119N also resulted in impaired DDR. Taken together, our results indicate that the naturally occurring S119N mutation causes abnormal DDR, hypersensitivity to IR, and enhanced genomic instability.

**Fig. 1:**
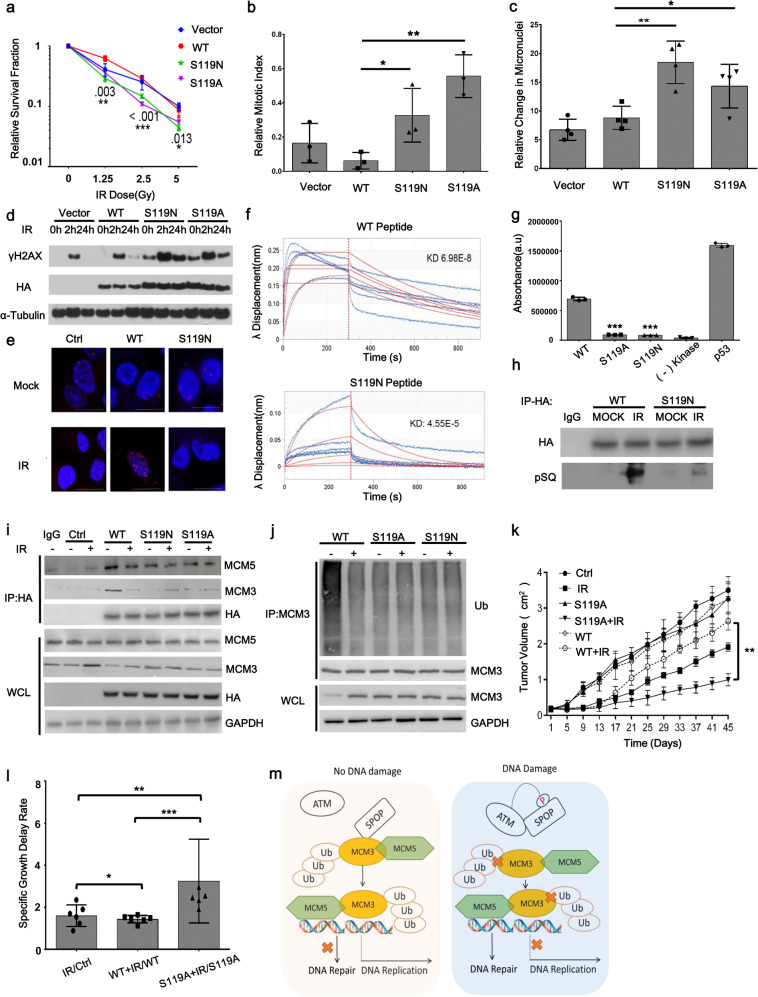
ATM-mediated SPOP Serine 119 phosphorylation is required for the DNA damage response in prostate cancer. **a** Cellular radiosensitivity was measured with the colony formation assay in LN-Cap cells. **b** Cell cycle analysis of PC-3 cells expressing the mutant SPOP. **c** Micronuclei quantification of PC-3 cells expressing the mutant SPOP, assessed by cell sorting. **d** Expression of γH2AX, HA, and α-Tubulin in PC-3 cells transiently transfected with HA-SPOP constructs, assessed by Western blotting. **e** In cell interaction was interrogated via the Proximity Ligation Assay. **f** In vitro interaction of ATM and SPOP was assessed by biolayer interferometry. **g** ATM Kinase activity was quantified by the in vitro kinase assay followed by measuring the production of ADP. **h** Pull-down of HA-SPOP by Co-IP in wild type and S119N cell lines after treated with 5Gy IR. **i** Pull-down of HA and MCM5 and MCM3 by co-immunoprecipitation. PC-3 cells transfected with vector, wild type, or the mutant SPOP constructs were treated with 5Gy of IR. **j** Immunoblotting of ubiquitination of MCM3 in SPOP WT, S119A and S119N knock-in cell lines. **k** Tumor volume of xenograft tumors post radiation. **l** Relative growth delay of tumors after radiation. **m** Schematic illustration of the model for the ATM-SPOP-MCM3 signaling in prostate cancer cells

Previously we found SPOP and ATM interacted in response to DNA damage.^4^ This endogenous interaction was confirmed in PC-3 cells (Supplementary Fig. S2a, b). To test if Serine 119 is required for this interaction, we used the In Situ Proximity Ligation Assay to check the interaction of ATM and SPOP. After IR, compared with the wild-type form, S119N mutation significantly abrogated the formation of foci in response to IR (Fig. 1e, Supplementary Fig. S2c), indicating that S119 is critical for the interaction of SPOP and ATM in response to IR. To test if the S119 mutation would interrupt binding directly in an in vitro system, we measured ATM-SPOP binding using biolayer interferometry. The wild-type peptide had roughly double the amount of peptide bound to ATM than the mutant peptide as measured by diffracted light wavelength (Fig. 1f). This indicated the S119N mutation increased dissociation with ATM.

To test the potential for direct phosphorylation, we performed an in vitro ATM kinase assay. We found that either S119N or Serine 119 mutated to alanine (S119A) caused a dramatic reduction in phosphorylation signals (Fig. 1g). To assess potential phosphorylation in cells, we pulled-down HA-tagged SPOP and probed with an anti-phospho-S/TQ antibody for ATM phosphorylation. We found that IR-induced SPOP S/TQ phosphorylation was inhibited by the ATM inhibitor KU55933, but not the ATR inhibitor AZD6738 or the DNAPK inhibitor AZD7648 (Supplementary Fig. S2d, e). In addition, we also knocked down ATM to examine SPOP phosphorylation (Fig. S2f). We found that, SPOP phosphorylation was significantly reduced in response to IR when ATM was knocked down. These data indicate that IR-induced SPOP S/TQ phosphorylation is ATM-dependent. Furthermore, S119A mutation abrogated phosphorylation (Fig. 1h). These data support the conclusion that ATM phosphorylates SPOP on Serine 119 in response to IR.

To further investigate SPOP’s function in the DDR, we aimed to identify proteins that SPOP interacted in response to IR. We performed the Stable Isotype Labeling by Amino acids in Cell culture (SILAC) assay, and found that proteins with increased SPOP interactions in response to IR are involved in cell division (Supplementary Fig. S3a). This indicates that SPOP might target these proteins for destruction as a part of the DDR to prevent cell cycle progression. Proteins with decreased SPOP interaction after radiation are mostly DNA duplex unwinding proteins (Supplementary Fig. S3b). This pathway suggests that SPOP dissociation with these proteins allow them to get access to DNA for appropriate damage sensing and repair.

Due to the phenotypes we observed in S119N and S119A mutated cells, we focused on validation of the interactions of SPOP with proteins that are significant to these processes. We selected Mini Chromosome Maintenance 5 (MCM5), a component of the MCM helicase complex for validation. However, despite the dissociation from the SPOP complex after IR, the MCM5 protein level did not change (Fig. 1i). However, we found that MCM3, a component of the MCM complex showed a decreased interaction with SPOP after IR (Fig. 1i, Supplementary Fig. S4a), and the MCM 3 protein level increased after IR. This change was abrogated when Serine 119 is mutated (Fig. 1i). We infer that IR-induced induction of MCM3 is dependent on SPOP S119 phosphorylation. We also found that the expression of MCM3 was enhanced in SPOP knock-down cells (Supplementary Fig. S4b). However, the increase of MCM3 in response to IR was no longer observed in the absence of SPOP (Supplementary Fig. S4b). In addition, we examined if degradation and ubiquitination of MCM3 was regulated by SPOP in response to DNA damage using the SPOP WT, SPOP S119A or S119N knock-in PC-3 cell lines. As shown in Fig. 1j, SPOP Serine 119 mutants (S119A or S119N) suppressed SPOP-induced MCM3 degradation and ubiquitination. In addition, as shown in Supplementary Fig. S4c, the IR-induced dissociation SPOP from MCM3 was abrogated in the absence of ATM. These results strongly support the conclusion that ATM-mediated SPOP Serine 119 phosphorylation is required for dissociation of SPOP-MCM3 and degradation of MCM3.

To strengthen the conclusion on the functional significance of ATM phosphorylation of Serine 119, we conducted radiosensitivity experiments for prostate cancer xenografts in athymic nude mice. We found that xenografts expressing the S119A mutant were significantly more sensitive as compared to wild-type or vector (Fig. 1k). The specific tumor growth delay rate for S119A is 2.7 while wild-type was 1.2 (*p* = 0.0001) (Fig. 1l). These results strongly support that the ATM phosphorylation of SPOP on Serine 119 is critical for reducing radiosensitivity.

In conclusion, we demonstrate that a prostate cancer-relevant mutation of SPOP on Serine 119 causes prolonged DNA repair and hypersensitivity to ionizing radiation. We prove that Serine 119 is required for the SPOP interaction with ATM, and demonstrate that ATM phosphorylates SPOP on Serine 119. Further, we identify the MCM5-MCM3 complex is among SPOP interacting proteins in response to DNA damage. We demonstrate that Serine 119 phosphorylation is required for the SPOP-MCM3 dissociation and it inhibits MCM3 ubiquitination and degradation (Fig. 1m). Taken together, we highlight a novel DDR pathway mediated by ATM phosphorylation of SPOP. These findings have clinical impact for prostate cancer patients with SPOP mutations, as DNA damaging therapies may be particularly effective in this subgroup. This also provides the first evidence for a pathophysiological relevant mutation linked to ATM phosphorylation in the DDR.

## Supplementary information

Supplemental information
