# Supplementary material for: A disease-relevant mutation of SPOP highlights functional significance of ATM-mediated DNA damage response
Source: Signal Transduct Target Ther. 2021 Jan 15;6:17. doi: 10.1038/s41392-020-00381-7 (PMC7809020; doi:10.1038/s41392-020-00381-7)
Supplement: Supplementary file 1 — Supplemental information [file 41392_2020_381_MOESM1_ESM.docx]

Supplementary Materials for

**A Disease-relevant Mutation of SPOP Highlights Functional Significance of ATM-mediated DNA Damage Response**

Mingming Xiao^1, †^, Joshua S. Fried^2, †^, Jinlu Ma^2,3, †^, Yang Su^1^, Rebecca J. Boohaker^2^, Qinghua Zeng^2^, Yaqi Mo^1^, Fanbiao Meng^1^, Rong Xiang^4,^* and Bo Xu^1, 2, 5,^*

^1^ Department of Biochemistry and Molecular Biology, Key Laboratory of Breast Cancer Prevention and Therapy, Ministry of Education, Tianjin Medical University Cancer Institute and Hospital, National Clinical Research Center for Cancer, Key Laboratory of Cancer Prevention and Therapy, Tianjin, Tianjin’s Clinical Research Center for Cancer, Tianjin 300060, China;

^2^ Department of Oncology, Southern Research Institute, Birmingham, AL 35205, USA, Cell Biology Program, University of Alabama at Birmingham, Birmingham, AL 35205, USA

^3^ Department of Radiation Oncology, First Affiliated Hospital, Xian Jiaotong University, Xi’an, China

^4^ Department of Biochemistry and Molecular Biology, Nankai University School of Medicine, Tianjin, China

^5^ Center for Intelligent Oncology, Chongqing University Cancer Hospital, Chongqing University School of Medicine, Chongqing, 400030, China

**^†^: Equal contribution**

***Correspondent Author:**

Bo Xu MD, PhD

Department of Molecular Radiation Oncology

Tianjin Medical University Cancer Institute and Hospital

West Huanhu Rd, Tianjin 300060, China

Email: [xubo@tmu.edu.cn](mailto:xubo@tmu.edu.cn); boxu2002@yahoo.com

Rong Xiang, PhD

Department of Biochemistry and Molecular Biology,

Nankai University School of Medicine, Tianjin, 300060, China

Email: rxiang@nankai.edu.cn

**This PDF file includes:**

Materials and Methods

Figures. S1 to S4

**Material and Methods**

**Cell culture**

Prostate cancer cell lines PC3，DU-145 and LNCaP cells were purchased from the American Type Culture Collection (ATCC, Manassas, VA, USA). PC-3 cells were grown in DMEM supplemented with 10% fetal bovine serum (FBS) and 5% penicillin/streptomycin (P/S). DU-145 and LNCaP cells were grown in RPMI with 10% FBS, 5% P/S and 5% L-glutamine. LNCaP cells with tetracycline-inducible HA-SPOP expression were graciously provided by Dr. Nick Mitsiades (Baylor School of Medicine) [22]. These cells were grown in RPMI with 10%FBS, 5% P/S, 5% L-glutamine, and 30 ug/mL G418 (Gibco, Dublin, Ireland). PWR1E and RWPE-1 prostate epithelial cell lines (ATCC) were grown in PREBM media supplemented with the nutrient bullet kit (Lonza, Mapleton, IL).

**Construction of SPOP S119A and S119N knock-in cell lines using Crisper-Cas9**

The sequence of exon 7 of the SPOP gene was screened for NGG proto-adjacent motif (PAM) sequences adjacent to the Ser119 coding region in humans. The sgRNA-target sites (5’- TCCCCACCCCAGAGAGTCAACGG -3’) with the best cutting efficiency and fewer predicted was selected by an algorithm. Vector loaded with the Cas9 gene and sgRNA targeting the promoter sequence of SPOP plasmid were co-transfected into PC-3 cells. Colonies with the heterozygous mutation were selected.

**Irradiation**

Ionizing radiation was delivered by an X-Rad 320 irradiator (Precision X-Ray Inc. N. Branford, CT, USA).

**Antibodies and plasmids**

Mouse anti-HA, and rabbit anti-GAPDH were purchased from Cell Signaling (Danvers, MA, USA). Rabbit anti-ATM, and rabbit anti-γH2AX antibodies were purchased from Abcam (Cambridge, MA, USA). Rabbit anti α-Tubulin was purchased from Sigma (St. Louis, MO, USA). The phospho-(Ser/Thr) ATM/ATR substrate antibody was purchased from Cell Signaling. Rabbit anti-Ub was purchased from Santa Cruz.

HA-tagged SPOP expression constructs including wild-type, Y87C, HA-F102C, S119N, W131G, F133L, F133V are all on the pcDNA 3.1 backbone and were provided by Dr. Nicholas Mitsiades. The serine 119 to alanine mutation (S119A) was generated by site directed mutagenesis.

The oligonucleotides for shControl and shSPOP constructs were synthesized and inserted into the pLKO.1 vector. The shRNA target sequence of human SPOP is 5′-CCGGCACAAGGCTATCTTAGCAGCTCTCGAGAGCTGCTAAGATAGCCTTGTGTTTTTTG-3′

**Transfection and induction of plasmids**

Transient transfections were done using Attractene transfection reagent (Qiagen, Hilden, Germany), with an appropriate amount of DNA and serum free OPTIMEM (Gibco, Dublin, Ireland). The transfection reagent was used at a 3:1 ratio with DNA. The amount of DNA, transfection reagent and media were adjusted based on the amount of cells being transfected. 16 hours following the addition of the transfection mix, the serum free media was replaced with media supplemented with an additional 10% FBS. LNCaP cells were induced to express HA-SPOP constructs by the addition of 200ng/mL of tetracycline to the media for 48 hours.

**Western Blotting**

Protein lysates were electrophoresed across an SDS gel, and transferred to a nitrocellulose membrane. The membrane was blocked in 5% milk in TBST, and probed overnight at 4ºC with appropriate antibodies. The membranes were then washed and probed for one hour at room temperature with secondary antibodies. Finally, membranes were incubated in developing solution (Thermo, Waltham, MA, USA) and developed onto film. Densitometry was done using Image J (National Institute of Health, Bethesda, MD, USA).

**Micronuclei Quantification**

The micronuclei staining kit was purchased from Intellicyt (Albuquerque, NM, USA). Cells were plated in 384-well plates at 3000 cells per well. The cells were then treated with radiation and fresh media was added 24 hours following radiation. 72-96 hours following radiation the cells were stained according to the protocol. Cell sorting was done with the IQue (Intellicyt). Analysis was done using Forecyt software (Intellicyt).

**Cell Cycle Analysis**

Transfected cells were dosed with radiation and harvested 90 minutes following radiation. The cells were fixed in methanol overnight at 4ºC. Fixed cells were then permeabilized with a solution containing 1% FBS and 1% triton-100 in PBS for 30 minutes. Permeabilized cells were probed with an anti-phospho H3 antibody conjugated to alexa fluor 488 (Cell Signaling). The cells were then washed and stained with propidium iodide (Molecular Probes, Eugene, OR, USA) for 30 minutes. Finally the cells were sorted via a FACS Caliber (Becton Dickinson, Franklin Lakes, NJ, USA). Analysis was done using flowjo software.

**Immunofluorescence**

Cells were fixed with 4% paraformaldehyde for 15min and permeabilized in 0.25% Triton X-100 for 10min, and incubated with the primary antibody diluted in 1% bovine serum albumin 1 h at room temperature or overnight at 4°C. Cells were then washed three times with PBS and incubated with a fluorescent-conjugated secondary antibody diluted in 1% bovine serum albumin for 1h at room temperature in the dark. DAPI diluted in 1× PBS was used to stain DNA. Slides were mounted using microscope immersion oil and visualized using a fluorescence microscope.

**3-(4,5-Dimethylthiazol-2-yl)-2,5-diphenyltetrazolium bromide (MTT)**

Transfected cells were treated with increasing doses of ionizing radiation or left untreated. 24 hours following radiation all cells were supplemented with fresh media. 72 hours following radiation MTT (Acros Organics, NJ, USA), was added to the media in a 1:10 ratio. The cells were then placed back in the incubator until the MTT had been metabolized. PBS was then added to the media in a 1:10 ratio and the plates were kept overnight at 4ºC while covered. Absorbance was read at 570nm by a Synergy 4 plate reader (Biotek, Winooski, VT, USA).

**Colony formation assay**

PC-3 cells were plated into six-well plates 24 hours after transfection in defined numbers. 24 hours following re-plating the cells were dosed with increased amounts of radiation. Fresh media was added after seven days. Once cell colonies had begun to reach 50 cells in size (10-14 days), colonies were fixed with 20% methanol. Following fixation the colonies were stained with crystal violet before the number of surviving colonies (>50cells) were counted.

**Co-immunoprecipitation (Co-IP)**

Cell lysates were pre cleared for four hours with a matrix from Santa Cruz. After pre clearing antibody was added along with species specific beads (Santa Cruz). The lysates were rotated overnight in 4ºC with the beads and antibody. The bead lysate mixture was washed once with PBS and spun down. The supernatant was then used for immunoblotting.

***In vitro* kinase assay**

*In vitro* kinase assay was done according to the protocol from Millipore. Briefly, 10 ng of purified constitutively active ATM (GenBank NM_000051) was incubated with 6 µM of wild type, S119N, or S119A SPOP peptide (Biomatik, Wilmington, DE, USA) of 10 amino acids in length or full length p53 in the presence of reaction buffer. 250 µM of ATP solution containing magnesium and manganese acetate was added to start the reaction. The reaction was allowed to proceed for one hour at room temperature. The reaction was stopped using ADP Glo reagents (Promega, Madison, WI, USA). Absorbance was read using a Synergy 4 plate reader (BioTek).

**In Situ Proximity Ligation Assay (in situ PLA)**

LNCaP cells were plated on to coverslips. Following induction cells were mock-treated or dosed with 5Gy of IR. Two hours following IR, coverslips were blocked and permeabilized with buffer from Duolink/Sigma. Then, cells were probed with mouse anti-HA and rabbit anti-ATM antibodies and stained with the Duolink PLA kit. Slides were imaged with confocal microscopy (Nikon, Tokyo, Japan). Cellular PLA foci, which denotes an interaction, were counted for each experimental condition from at least five different fields with more than 30 cells of view.

***In vitro* binding assay**

Binding assays were all conducted using biolayer interferometry (BLI) on an OctetRed (Pall ForteBio, Menlopark, CA, USA) with Protein A labeled dip-and-read biosensors. Recombinant constitutively active ATM was loaded onto the biosensors at a concentration of 2ng/µl. Loading was done for 300 seconds (s), followed by a baseline reading then an association reading for 300s followed by a 600s dissociation reading. SPOP peptide concentration ranged from 0.740 to 60uM in 3:1 serial dilutions for initial binding experiments. Binding data was analyzed using the Octet software analysis system.

**Stable Isotope Labeling of Amino Acids in Cell Culture (SILAC)**

Lysate preparation: HA-tagged SPOP was expressed in DU-145 cells. Cells were grown for 5-6 generations in “heavy” medium. In parallel, untransfected DU145 cells were grown in “light” medium. HA-SPOP-DU145 cells were dosed with 2 Gy IR. 2 hours following IR, cell monolayers from both heavy and light cultures were washed once with PBS and scraped into RIPA buffer at 0-4°C. The cell lysates from heavy and light cells were clarified by centrifugation for 15 minutes at 15,000 rpm and equivalent amounts of extract (determined by BCA assay, Pierce, Waltham, MA, USA) were added to yield approximately 4-5mg of total protein/mL and a final volume of 4mL.

Affinity beads: 500μl of HA agarose beads (Santa Cruz) suspension was washed once with medium salt (MS) buffer and mixed with 1mL of lysate (~ 5-7mg/mL) from light DU145 lysate. The beads were incubated for 1hr at room temperature and washed twice with MS buffer and once with high salt (HS). They were then resuspended in the original volume of MS buffer and used with the heavy/light lysate prepared as above.

Affinity purification: 100μL of pre-blocked HA agarose bead suspension was then added to 4mL of the heavy/light mixed lysates followed by rotation at 4°C for 2hr. The beads were centrifuged for 2 minutes at 2000 rpm and washed once with 15-20 volumes of 1 x CSK-NP and twice with 15-20 volumes of MS buffer. This was followed by resuspension of the beads in 300μL of HS buffer for 20 minutes at 0-4°C and centrifugation. Elution of the bait was carried out by incubation of the beads with 150-200μL of HA elution buffer. The supernatant is the HA-peptide eluate fraction (HA peptide fraction). Both, HS and HA-peptide fractions were subjected to MS analysis.

**Mouse xenografts and lentivirus injection**

Athymic nude mice (4 weeks old) were purchased from the Beijing Experimental Animal Center and maintained in a specific pathogen–free facility approved by the Laboratory Animal Center of Xi’An Jiaotong University School of Medicine. All animal studies were performed following the animal procedures approved by Institutional Animal Care and Use Committee (IACUC) of Xi’An Jiaotong University, which is consistent with national regulatory standards. For radiation re-growth delay studies, 1×10^7^ LNCaP cells (mixed with Matrigel at a volume ratio of 1:1) were injected subcutaneously in the flank of the 4-week-old athymic nude mice. Tumor growth was observed every second day until the diameter of tumor reached 0.6 to 0.8 mm as measured by caliper. At this point animals were randomized into 8 groups (6 mice/group): control; IR; SPOP wt; SPOP S119A; SPOP S119N; SPOP wt + IR; SPOP S119A + IR and SPOP S119N + IR. Radiation treatment consisted of 10-Gy for 1 fraction. The mice were treated with lentiviruses respectively by carefully pipetting it on top of the epidermis covering the tumors in 100 µl DMEM, lentivirus was administered twice per week. Three days post-injection, the mice were exposed to radiation. The length and width of the treated tumors were measured using a Vernier caliper every two days. Tumor size was measured with calipers using the formula V= (a × b^2^)/2, in which *a* and *b* are the largest and the smallest perpendicular diameters, respectively. Tumors were followed individually until they measured greater than 800 mm^3^. The mean growth delay for each treatment group was calculated as the number of days for the mean of the treated tumors to grow to 800 mm^3^ divided by the number of days for the mean of the control group to reach the same size.


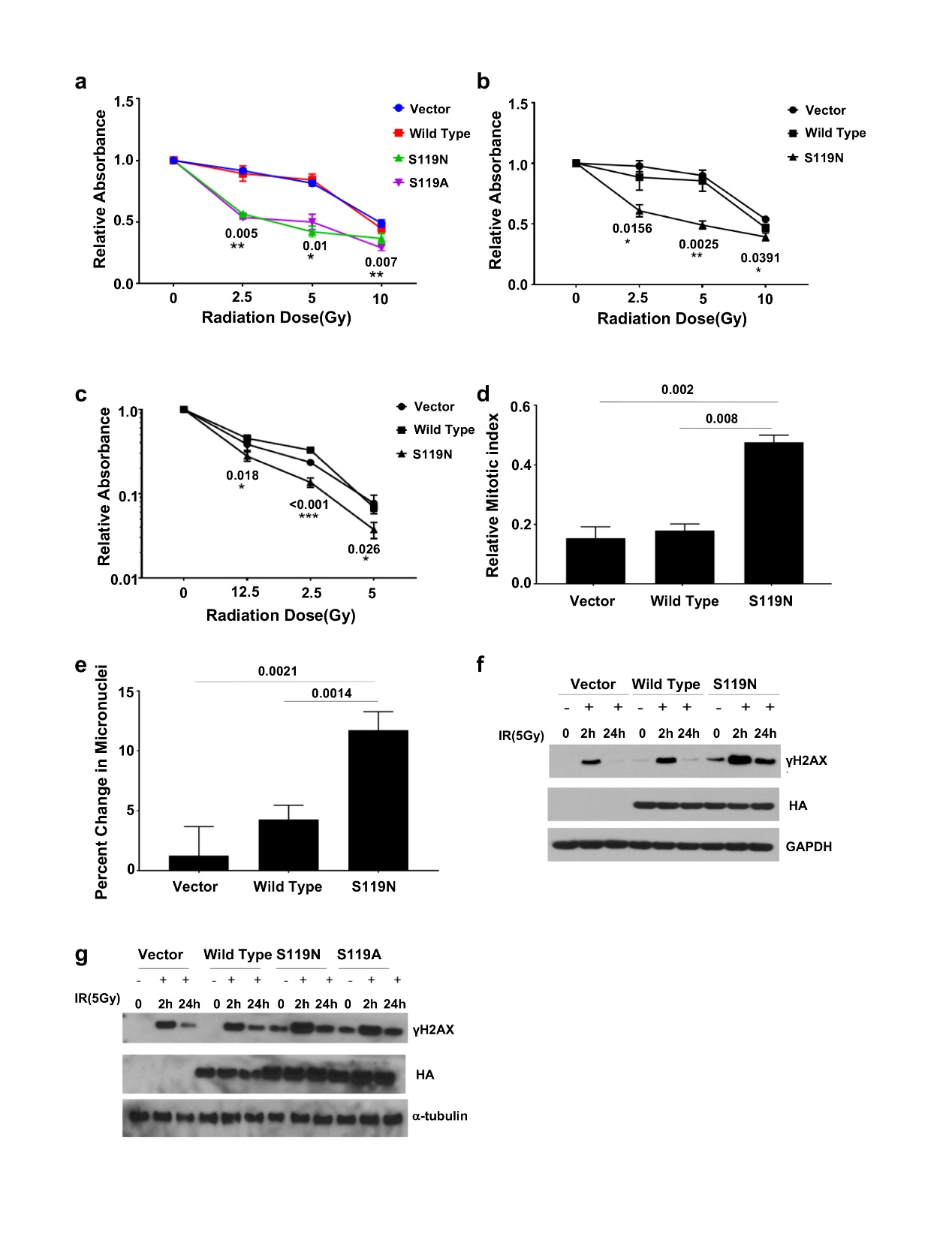


**Supplementary Fig. S1. SPOP Serine 119 mutation causes defects in the DDR.** (a) PC-3 cells transiently expressing vector only, wild-type or S119N of SPOP were irradiated and assessed via MTT assay. (b)Cell viability was assessed via MTT assay in LNCaP cells. (c) Radiosensitivity was measured with the colony formation assay in LNCaP cells. (d) Cell cycle analysis of LNCaP cells. (e) Micronuclei quantification of cells, assessed by cell sorting. (f) Expression of γH2AX, HA, and GAPDH in LNCaP cells induced to expressed HA-SPOP constructs by tetracycline, assessed by western blotting. (g) Expression of γH2AX, HA, and GAPDH in PWR1E cells transiently transfected
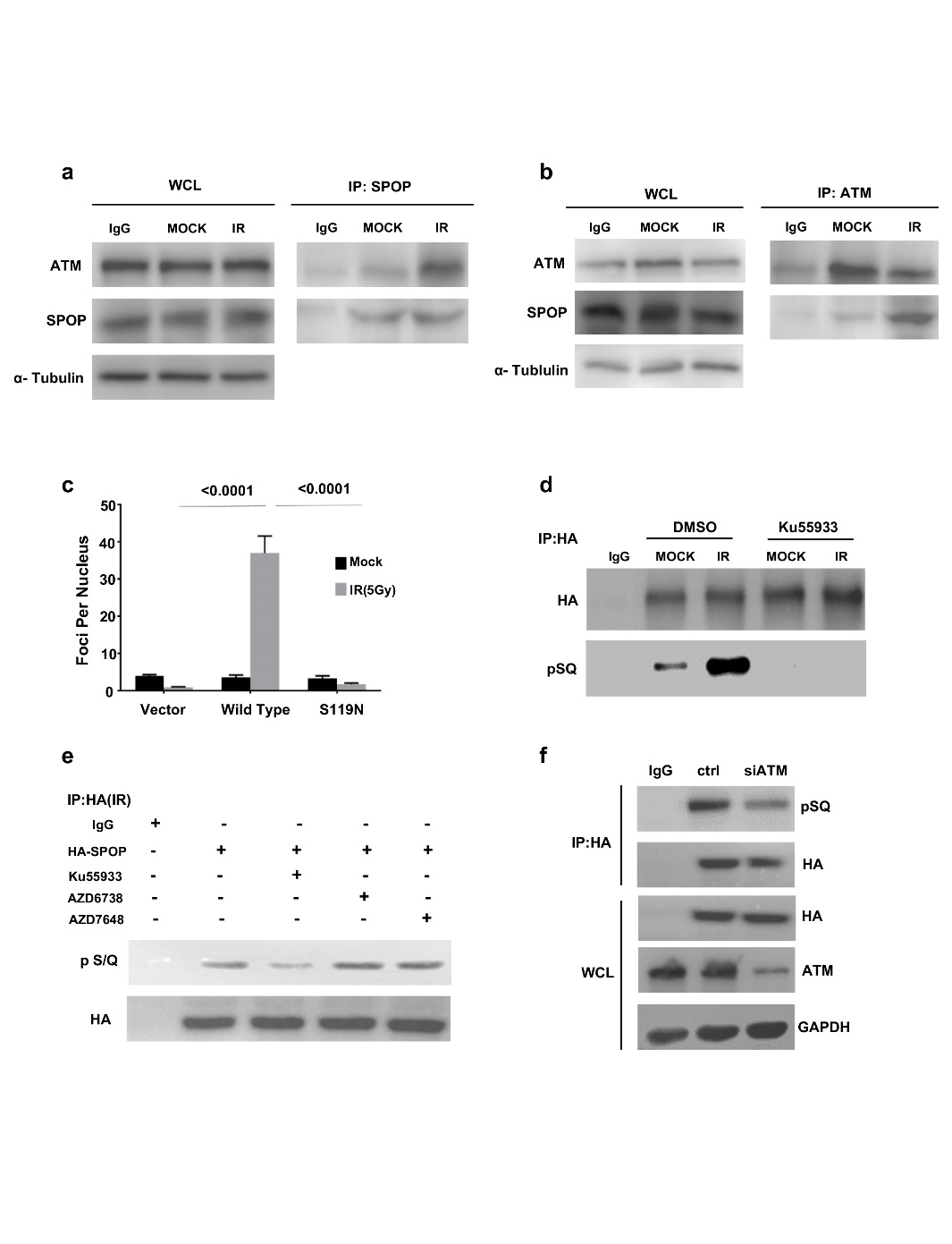
with HA-SPOP constructs, assessed by western blotting.

**Supplementary Fig. S2. SPOP is phosphorylated on Serine 119 after radiation in an ATM dependent manner.** Endogenous SPOP (in a) or ATM (in b) was immunoprecipitated in cells 2 h after treating with mock or IR (6 Gy). The immunoprecipitates were subjected to immunoblotting using indicated antibodies. Total cell lysates were included as loading controls. (c) In cell interaction was interrogated via Proximity Ligation Assay. Foci were counted if they co localized with DAPI staining. (d) Pull-down of HA SPOP by Co-IP. Transfected PC3 cells were treated with radiation in the presence or absence of an ATM inhibitor. (e) Pull-down of HA-SPOP by Co-IP. Transfected PC3 cells were treated with IR in the presence or absence of the ATM, ATR and DNAPKinhibitors. (f) Pull-down of HA-SPOP by Co-IP. PC3 cells transfected
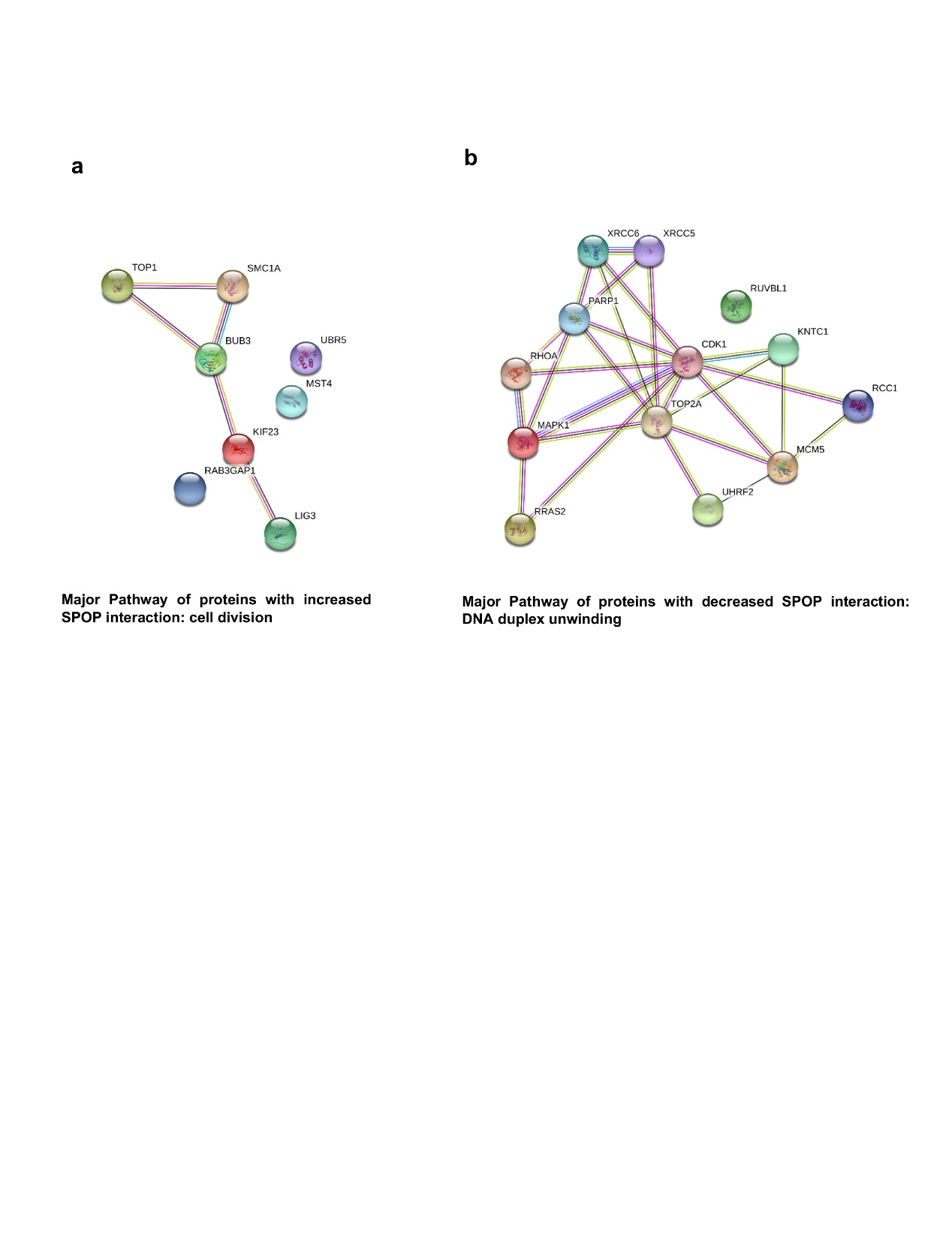
with control or ATM siRNA.

**Supplementary Fig. S3. DNA damage induces a SPOP complex that involves DNA repair and cell cycle regulatory proteins.** Protein interaction / pathway analysis was done using https://string-db.org/. Proteins from mass spectrometry analysis were grouped based on how interaction with SPOP was altered by DNA damage. (a) Shows proteins with increased interaction after
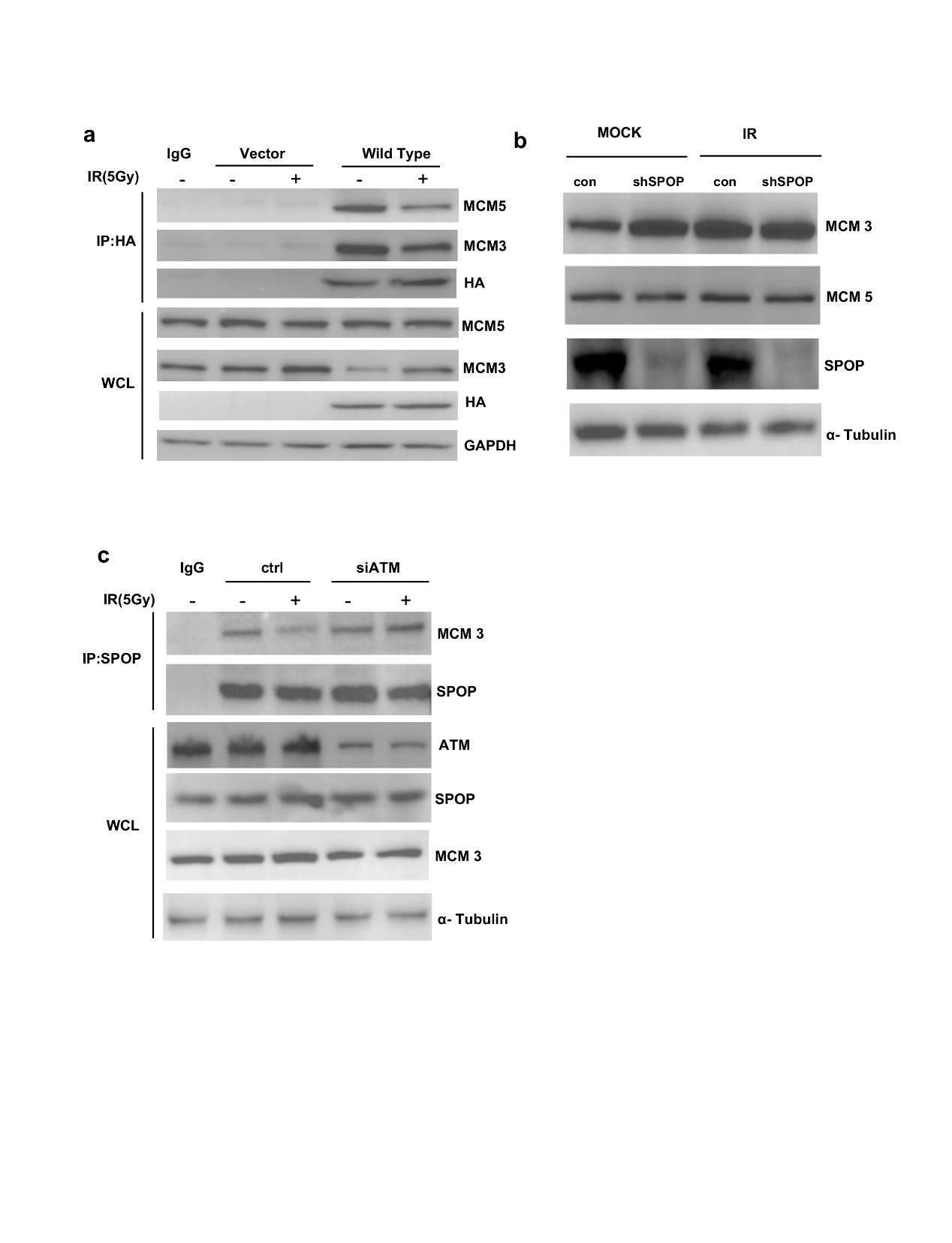
radiation. (b) shows proteins with decreased interaction after radiation.

**Supplementary Fig. S4. SPOP interaction with MCM5 and MCM3 in response to IR.** (a) Pull-down of HA and MCM5 and MCM3 by co-immunoprecipitation. PC-3 cells transfected with vector, Wild Type were treated with 5Gy of IR. (b) Immunoblotting of MCM3 and MCM5 in PC-3 cells transfected with either control or SPOP shRNA in the presence or absence of IR. (c) Pull-down of SPOP and MCM3 by co-immunoprecipitation. PC-3 cells transfected with scrambled control siRNA or ATM siRNA were mock-treated or treated with 5Gy of IR.
